# Replication characteristics of equine herpesvirus 1 and equine herpesvirus 3: comparative analysis using ex vivo tissue cultures

**DOI:** 10.1186/s13567-016-0305-5

**Published:** 2016-01-15

**Authors:** Haileleul Negussie, Yewei Li, Tesfaye Sisay Tessema, Hans J. Nauwynck

**Affiliations:** Laboratory of Virology, Department of Virology, Parasitology and Immunology, Faculty of Veterinary Medicine, Ghent University, Salisburylaan 133, 9820 Merelbeke, Belgium; College of Veterinary Medicine and Agriculture, Addis Ababa University, P.O. Box 34, Debre Ziet, Ethiopia; Institute of Biotechnology, College of Natural and Computational Sciences, Addis Ababa University, P.O. Box 1176, Addis Ababa, Ethiopia

## Abstract

Replication kinetics and invasion characteristics of equine herpesvirus-1 and -3 (EHV-1/-3) in nasal and vaginal mucosae were compared using explants. The explants were cultured during 96 h with little change in viability. The tissues were inoculated with EHV-1 03P37 (neuropathogenic), 97P70 (abortigenic) and EHV-3 04P57, collected at 0, 24, 48 and 72 h post inoculation (pi) and stained for viral antigens. Both EHV-1 and EHV-3 replicated in a plaquewise manner. The plaques were already observed at 24 h pi, their size increased over time and did not directly cross the basement membrane (BM). However, EHV-1 infected the monocytic cells (MC) and hijacked these cells to invade the lamina propria. In contrast, EHV-3 replication was fully restricted to epithelial cells; the virus did not breach the BM via a direct cell-to-cell spread nor used infected MC. EHV-1-induced plaques were larger in nasal mucosa compared to vaginal mucosa. The opposite was found for EHV-3-induced plaques. Both EHV-1 strains replicated with comparable kinetics in nasal mucosa. However, the extent of replication of the abortigenic strain in vaginal mucosa was significantly higher than that of the neuropathogenic strain. Two-to-five-fold lower numbers of EHV-1-infected MC underneath the BM were found in vaginal mucosa than in nasal mucosa. Our study has shown that (i) EHV-1 has developed in evolution a predisposition for respiratory mucosa and EHV-3 for vaginal mucosa, (ii) abortigenic EHV-1 replicates better in vaginal mucosa than neuropathogenic EHV-1 and (iii) EHV-3 demonstrated a strict epithelial tropism whereas EHV-1 in addition hijacked MC to invade the lamina propria.

## Introduction

Equine herpesvirus-1 (EHV-1) and equine herpesvirus-3 (EHV-3) cause contagious diseases in equids worldwide [1, 2]. EHV-1 is responsible for respiratory disorders, abortion, neonatal foal death, myeloencephalopathy or chorioretinopathy [1, 3]. EHV-3 is the cause of equine coital exanthema (ECE), a genital disease that is transmitted venereally. This disease is characterized by the development of papules, vesicles, pustules and ulcers in the mucosa of the vagina and vestibula of mares and the penis and prepuce of stallions, in the skin of the perineal region of the mares and occasionally on the skin of the lips and mucosa of the upper respiratory tract [2, 4, 5]. Both EHV-1 and EHV-3 are members of the subfamily *Alphaherpesvirinae* with about 150 kilobases double-stranded DNA genome, consisting of 76 unique open reading frames [6, 7]. However, antigenically, genetically and pathogenetically, EHV-1 and EHV-3 are significantly different [2].

Latently infected equines are important biological reservoirs for EHV-1 [8, 9] and EHV-3 [2, 10]. The periodic virus reactivation from latency leads to the production of infectious virus that serves as a source of infection [1, 10]. EHV-1 is transmitted to susceptible equids through direct contact with virus-laden respiratory secretions or indirectly with fomites [1]. Although EHV-3 is primarily transmitted through coitus, contaminated fomites have also been implicated in its spread [11].

After initial infection, EHV-1 replicates in mucosal epithelial cells of the upper respiratory tract and causes erosions and viral shedding into the environment [1, 12]. The virus then invades the underlying lamina propria by infected immune cells [12–14]. Hereafter, EHV-1 disseminates throughout the body using infected mononuclear cells as Trojan horses. The cell-associated viremia allows the virus to arrive and replicate at the endothelial cells of the target organs, which leads to vasculitis and ischemic thrombosis [15]. In contrast, EHV-3 replicates in the stratified epithelium of epidermal tissues present at the mucocutaneous margins and skin [2]. Destruction of the epithelium by the lytic virus infection elicits a vigorous, localized inflammatory response that gives rise to the formation of characteristic cutaneous lesions of ECE [2]. Infertility and abortion associated with EHV-3 have not been reported [16, 17]. However, the disease has a negative impact in the equine industry as a result of the forced, temporary disruption of the mating activities of affected stallions and mares [2, 18].

Different strains of EHV-1 have a different pathological outcome, which is correlated with the variation in the ability to disseminate and establish infection at vascular endothelial cells of the target organs such as the endometrium, the central nervous system and the eye [3, 19]. The respiratory mucosal surface plays a major role in EHV-1 primary replication and transmission [13]. Variation in the structural barriers, microenvironment and the composition of available target cells on mucosal tissues may dramatically influence the efficiency of EHV-1 replication. Previous studies have shown the invasion mechanisms of EHV-1 in the equine respiratory mucosa using nasal explants [20] and an in vivo experiment [14]. It was demonstrated that during infection of epithelial cells, EHV-1 is infecting mucosal monocytes and is hijacking these cells to invade the deeper connective tissues. Despite these studies, little is known about the replication efficiency of EHV-1 strains in the vaginal epithelial mucosa, which could serve as an alternative EHV-1 portal of entry.

EHV-3 is highly host specific. It replicates only in cell lines derived from equids and a laboratory animal model has not been identified for EHV-3 infection [2, 21]. To date, experimental studies with EHV-3 have solely been done in the natural hosts, equids. To study early events of EHV-3 mucosal invasion, an alternative in vitro model would be very valuable. Previously, the replication characteristics of other equine alphaherpesviruses, EHV-1 and EHV-4, have been studied in respiratory mucosa explants [20, 22]. The replication of EHV-4 was restricted to the epithelial cells, whereas EHV-1 was also infecting mucosal leukocytes. However, the replication kinetics and the invasion characteristics of EHV-3 in mucosae are still not known.

Therefore, this study was designed to compare the replication kinetics and the invasion characteristics of the neuropathogenic and abortigenic strains of EHV-1 and EHV-3 in the nasal and vaginal mucosae using ex vivo tissue cultures.

## Materials and methods

### Tissue collection

The nasal and vaginal tissues were obtained from healthy horses with an estimated age of 5–10 years after slaughter in the abattoir. The absence of recent EHV-1 and EHV-3 infection was demonstrated by the absence of cytopathic effect up on inoculation of rabbit kidney 13 cell and equine dermal cell lines with tissue suspensions (10% W/V). A complement-dependent seroneutralization test was also performed on the serum to determine EHV-specific antibody titres. The stage of the reproductive cycle was also evaluated by visual inspection of the ovaries. All the animals were found to be in diestrus.

All the tissues were collected with transport medium containing phosphate buffered saline (PBS) supplemented with 1 μg/mL gentamycin (Invitrogen, Paisley, UK), 1 mg/mL streptomycin (Certa, Braine l’Alleud, Belgium), 1 mg/mL kanamycin (Sigma, St. Louis, MO, USA), 1000 U/mL penicillin (Continental Pharma, Puurs, Belgium) and 5 μg/mL amphotericin B (Bristol-Myers Squibb, New York, USA). All collected tissues were placed in a cooler containing ice for transport to the laboratory and were processed immediately after arrival. The mucosae were stripped from the underlying tissues and dissected into explants of approximately 25 mm^2^.

### Air–liquid culture model

Cultivation of the explants was performed according to the protocol as previously described [23, 24] with some modifications. Briefly, the explants were cultured using fine-meshed gauze, on 6-well tissue-culture plate, in an air–liquid interface with epithelium facing upwards. The explants were cultured with serum free medium consisting of a 1:1 mixture of Roswell Park Memorial Institute medium (RPMI GlutaMAX™) (Invitrogen) and Dulbecco’s Modified Eagle’s Medium (DMEM GlutaMAX™) (Invitrogen) supplemented with 1 μg/mL gentamycin (Invitrogen), 0.1 mg/mL streptomycin (Certa) and 100 U/mL penicillin (Continental Pharma). To mimic an air-interface as in the living animals, the explants were covered with a thin-film of medium and maintained at 37 °C in an atmosphere containing 5% CO_2_.

### Evaluation of tissue viability

Tissue viability was monitored by evaluating ciliary beating of the epithelial cells of the nasal explants using light microscope and by quantifying the apoptotic cells using in situ cell death detection kit (Roche Diagnostics Corporation, Basel, Switzerland). Terminal deoxynucleotidyl transferase mediated dUTP nick end labeling (TUNEL), preferentially labels DNA strand breaks generated during apoptosis. The test was performed according to the manufacturer’s guidelines on cryosections preserved in methocel^®^ (Sigma) at 0, 24, 48, 72 and 96 h of cultivation. In both the epithelium and lamina propria, the percentage of TUNEL-positive cells was quantified in five randomly chosen fields of 100 cells each. TUNEL-positive cells were detected and enumerated by fluorescence microscopy (Leica DMRBE, Wild Leitz GmbH, Heidelberg, Germany).

### Virus strains used for infections of the explants

Two Belgian EHV-1 strains, representing neuropathogenic and abortigenic variants of EHV-1, were used in this study. These strains were typed by sequencing the DNA polymerase gene of EHV-1 as previously described [25]. The abortigenic EHV-1 strain 97P70 which was isolated from an aborted fetus in 1997 and the neuropathogenic strain 03P37 which was isolated from the peripheral blood mononuclear cells of paralytic horses in 2003, were used. EHV-1 virus stocks used for inoculation of the explants were at the 6th passage; four passages in equine embryonic lung cells and two subsequent passages in rabbit kidney 13 cells.

The EHV-3 strain 04P57 which was isolated from a horse with typical ECE lesions in Belgium in 2004, was used in this experiment. The virus stock used for inoculation was at the second passage in equine embryonic kidney cells. Genetic and pathogenic differences between EHV-3 strains have not been described and therefore, only one EHV-3 strain was included in this study.

### Inoculation of the explants

After 24 h of culture, explants were inoculated with the strains of EHV-1 and EHV-3 by submerging the tissue in 1 mL of inoculum containing 10^6.5^ TCID_50_ for 1 h at 37 °C and 5% CO_2_. After incubation, explants were washed twice with warm medium and transferred back to the original 6-well plates containing gauze and medium. At 0, 24, 48 and 72 h post inoculation (pi), explants were collected, embedded in methylcellulose medium (Methocel^®^ MC, Sigma-Aldrich, St. Louis, USA) and frozen at −70 °C.

### Immunofluorescence staining and plaque analysis

A double immunofluorescence staining was performed to detect and localize EHV-1 and EHV-3-infected cells at 0, 24, 48 and 72 h pi in 100 consecutive 16 µm cryosections of the explants. The cryosections were fixed with 100% methanol for 20 min at −20 °C. The basement membrane (BM) of the tissues was stained with monoclonal mouse anti-collagen VII antibodies (Sigma-Aldrich), followed by Texas red-labeled goat anti-mouse antibody (Invitrogen). Biotinylated equine polyclonal anti-EHV-1 IgG antibodies [26] were used to label the EHV-1 viral antigens and biotinylated rabbit polyclonal anti-EHV-3 IgG antibodies to label EHV-3 viral antigens. Next, fluorescein isothiocyanate (FITC) labeled streptavidin (Invitrogen) was added. Mock-inoculated cryosections were stained as negative controls. In each steps, cryosections were incubated at 37 °C for 1 h and were washed afterwards three times with PBS. Hoechst 33342^®^ staining (molecular probes) was performed to visualize the nuclei of the cells. Then, the cryosections were mounted with glycerol containing antifading agent 1, 4-Diazobicyclo-(2, 2, 2-octane (DABCO^®^). The plaques were visualized using a confocal fluorescence microscope (Leica DMRBE, Wild Leitz GmbH, Heidelberg, Germany). The number of plaques per 8 mm^2^ explants and the plaques size were quantified using Leica LAS AF Lite software. The plaques on the borders and edge of the explants were excluded from analysis.

### Identification and quantification of single infected cells

To identify and quantify EHV-1 and EHV-3 infected single cells, a double immunofluorescence staining was performed. At each collection time point, 10 μm-thick cryosections of tissue explants were fixed in 100% methanol at −20 °C for 20 min. For each tissue and each time point, 20 cryosections were stained for each cell surface marker separately. Monoclonal antibodies DH59B (VMRD, USA), UC F6G-3 (California University, Davis, USA) and 1.9/3.2 (VMRD, USA) were used as markers for CD172a cells of the monocyte lineage, CD3 cells (pan T-lymphocytes) and IgM cells (B-lymphocytes), respectively. Then, the cryosections were incubated with Texas Red^®^-labeled goat anti-mouse IgG antibodies (Invitrogen). In the second step, EHV-1 and EHV-3 viral proteins were stained with biotinylated equine polyclonal anti-EHV-1 IgG antibodies [26] and biotinylated rabbit polyclonal anti-EHV-3 IgG antibodies, respectively, followed by streptavidin-FITC (Invitrogen). Sections of mock-inoculated explants and isotype matched irrelevant control antibodies were used as negative controls. In each step, cryosections were incubated at 37 °C for 1 h and washed three times with PBS. The nuclei were counterstained with Hoechst 33342^®^ for 10 min. At each time point, the percentage of EHV-1 infected cells that are marker positive cells were calculated from 20 cryosections of 10 µm thick for each specific marker. All the cryosections were analyzed by confocal microscopy (Leica DMRBE, Wild Leitz GmbH, Heidelberg, Germany).

### Statistical analysis

The data were analyzed using SPSS version 20 software (SPSS Inc, Chicago, USA). Differences between the strains, tissues and among time points were compared by the analysis of variance (ANOVA) with post hoc multiple comparisons. Mann–Whitney test was also used as non-parametric test. All data are expressed as means with standard deviation (SD) of three independent experiments. Differences were considered statistically significant when *P* value was <0.05.

## Results

### Tissue viability

The viability of the cells in the nasal and vaginal explants were evaluated using TUNEL staining to detect DNA fragmentation associated with apoptotic cell death at 0, 24, 48, 72 and 96 h of cultivation. During the ex vivo tissue cultivation, the number of apoptotic cells in the epithelium slightly, but not significantly, increased over time. The percentage of TUNEL positive cells in the epithelium of the nasal and vaginal mucosae was 1.7 ± 0.2 and 1.9 ± 0.1, respectively, at 96 h of cultivation (Table 1). In the epithelium of the nasal mucosa, ciliary beating was observed during the whole experiment (up to 96 h of cultivation).

**Table 1:** **Percentage of TUNEL-positive cells in the epithelium and lamina propria of the nasal and vagina mucosae at different time points of cultivation.**

	Percentage of TUNEL-positive cells at indicated time points of cultivation				
	0 h	24 h	48 h	72 h	96 h
Nasal mucosa					
Epithelium	0.3 ± 0.1	0.4 ± 0.2	0.7 ± 0.1	1.3 ± 0.1	1.7 ± 0.2
Lamina propria	0.7 ± 0.1	1.1 ± 0.3	2.5 ± 0.3	3.1 ± 0.2	4.2 ± 0.2
Vaginal mucosa					
Epithelium	0.4 ± 0.2	0.7 ± 0.1	1.4 ± 0.3	1.7 ± 0.1	1.9 ± 0.1
Lamina propria	0.9 ± 0.2	1.3 ± 0.4	3.4 ± 0.5	3.6 ± 0.6	6.2 ± 0.4

### Invasion characteristics

**Figure 1:**
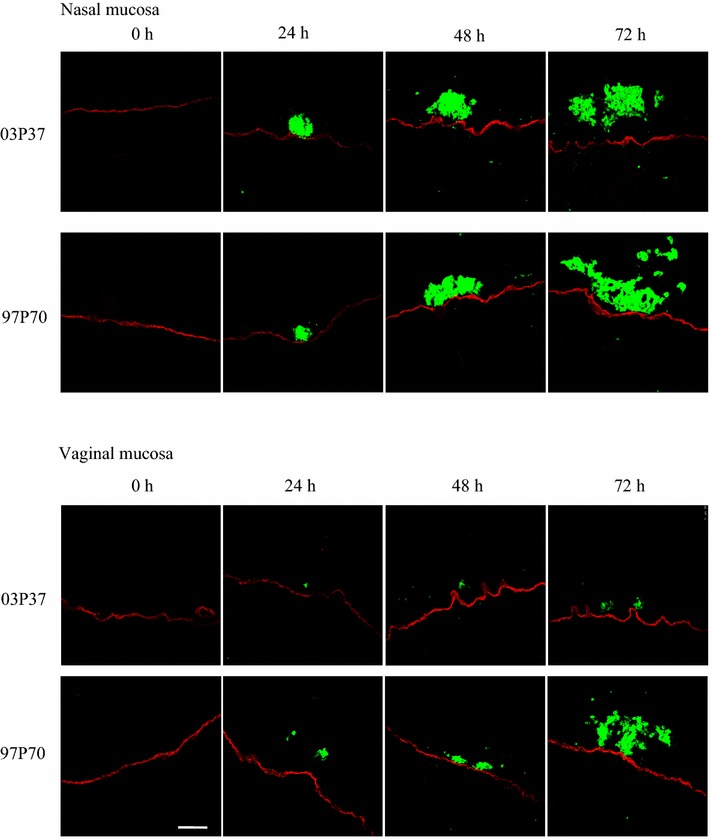
**Confocal photomicrographs showing EHV-1-induced plaques in nasal and vaginal explants inoculated with EHV-1 03P37 and EHV-1 97P70**. The BM is visualized using mouse anti-collagen VII and goat anti-mouse Texas Red^®^ antibodies. The viral antigens are detected using biotinylated equine polyclonal anti-EHV-1 IgG antibodies and striptavidin-FITC^®^. Scale bar 100 µm.

**Figure 2:**
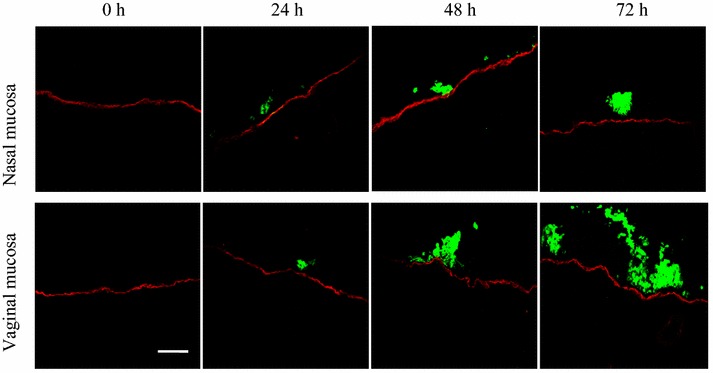
**Confocal photomicrographs showing EHV-3-induced plaques in the nasal and vaginal mucosa infected with EHV-3 04P57.** The BM is visualized using mouse anti-collagen VII and goat anti-mouse Texas Red^®^ antibodies. The viral antigens are detected using biotinylated rabbit polyclonal anti-EHV-3 IgG antibodies and streptavidin-FITC^®^. Scale bar 100 µm.

The invasion characteristics of EHV-1 and EHV-3 were assessed in the nasal and vaginal mucosae at 0, 24, 48 and 72 h pi. In mock-infected explants, plaques were not observed throughout the experiments. Both strains of EHV-l and EHV-3 replicated in a plaquewise manner and spread laterally on the epithelium. The plaques in the epithelium were already found at 24 h pi, their sizes significantly increased over time and did not cross the BM at all the time points pi. However, EHV-1 and EHV-3 exhibited different invasion characteristics. EHV-1 infects mononuclear immune cells to invade the lamina propria (Figure 1). In contrast, EHV-3 replication was restricted to the epithelium of the nasal and vaginal mucosae, where the virus neither breaches the BM nor infects individual monocytic immune cells at all time points pi (Figure 2).

### Number of plaques

**Figure 3:**
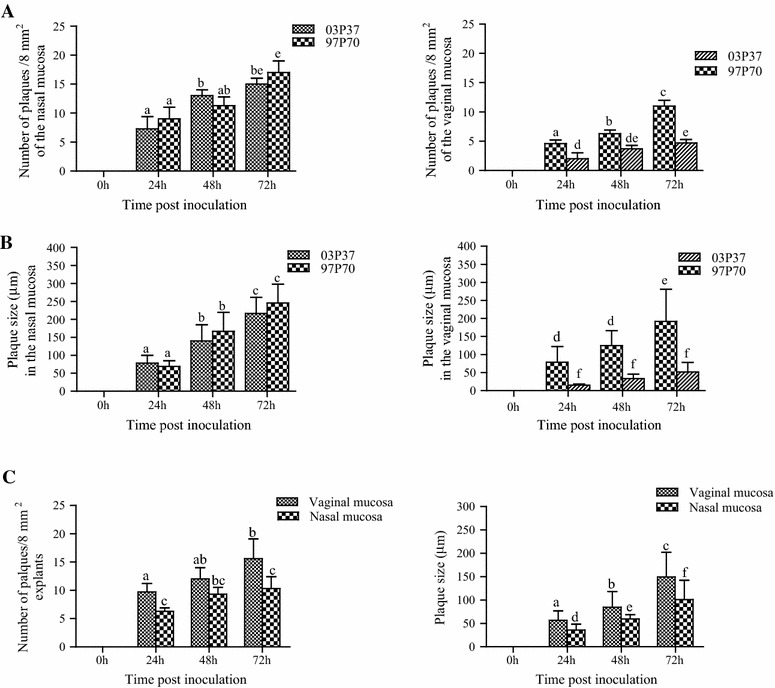
**Replication kinetics of EHV-1 (A and B) and EHV-3 (C) in the nasal and vaginal explants.** The number of plaques/8 mm^2^ explants and the plaque size are shown. Data represent mean ± SD of triplicate independent experiments. Significant differences are indicated by the use of different letters.

The number of plaques induced by EHV-1 and EHV-3 on 8 mm^2^ of explants was counted at 24, 48 and 72 h pi. In both mucosae, the average number of plaques induced by both strains of EHV-1 significantly increased (*P* < 0.05) over time. In the nasal mucosa, no significant difference (*P* > 0.05) was observed in the average number of plaques between both EHV-1 strains (Figure 3). However, the average number of plaques in the vaginal mucosa was significantly higher (*P* < 0.05) with the abortigenic strain 97P70 compared to the neuropathogenic strain 03P37 at all time points pi. Overall, the average number of plaques counted in the nasal mucosa was significantly higher (*P* < 0.05) than in the vaginal mucosa.

With EHV-3, the average number of plaques in the nasal mucosa slightly, but not significantly (*P* > 0.05) increased over time. However, the average number of plaques in the vaginal mucosa significantly increased (*P* < 0.05) between 24 and 72 h pi. Overall, the average number of plaques was significantly higher (*P* < 0.05) in the vaginal mucosa than in the nasal mucosa at 24 and 72 h pi (Figure 3).

### Plaques size

Both strains of EHV-1 had a different potential to spread from cell-to-cell in the nasal and vaginal mucosae. In the nasal mucosa, the neuropathogenic EHV-1-induced plaques enlarged significantly (*P* < 0.05) over time with an average of 77.9 ± 22.1 µm at 24 h pi, 140.1 ± 45.0 µm at 48 h pi and 216.1 ± 44.9 µm at 72 h pi. Likewise, the size of the plaques induced by the abortigenic strain significantly (*P* < 0.05) increased over time with an average of 68.9 ± 15.8 µm at 24 h pi, 166.9 ± 52.3 µm at 48 h pi and 245.5 ± 52.3 µm at 72 h pi. However, the average sizes of the plaques were not significantly different (*P* > 0.05) between both EHV-1 pathotypes (Figure 3). In the vaginal explants, the size of the plaques induced by the abortigenic strain significantly increased (*P* < 0.05) between 48 and 72 h pi with an average of 125 ± 41.7 µm and 192 ± 89.5 µm, respectively. Similarly, the plaque size slightly, but not significantly (*P* > 0.05) increased over time with the neuropathogenic strain. In contrast, the average size of the plaques induced by the neuropathogenic strain was significantly smaller (*P* < 0.05) when compared to the abortigenic strain at all time points pi (Figure 3).

Overall, the average sizes of the plaques induced by both EHV-1 strains were significantly larger (*P* < 0.05) in the nasal mucosa than in the vaginal mucosa.

With EHV-3, the average size of the plaques significantly increased (*P* < 0.05) over time in both the nasal and vaginal explants. The average size of EHV-3-induced plaques in the vaginal mucosa was 56.5 ± 20.1 µm, 84.5 ± 33.8 µm and 149.2 ± 53.0 µm at 24, 48 and 72 h pi, respectively. Similarly, an average of 35.7 ± 12.5 µm (24 h pi), 59.6 ± 9.1 µm (48 h pi) and 100.8 ± 41.5 µm (72 h pi) plaque size was recorded in the nasal mucosa. Overall, the average size of the plaques induced by EHV-3 was significantly higher (*P* < 0.05) in the vaginal mucosa, compared to the nasal mucosa at all time points pi (Figure 3).

### Identification and quantification of single infected cells

**Figure 4:**
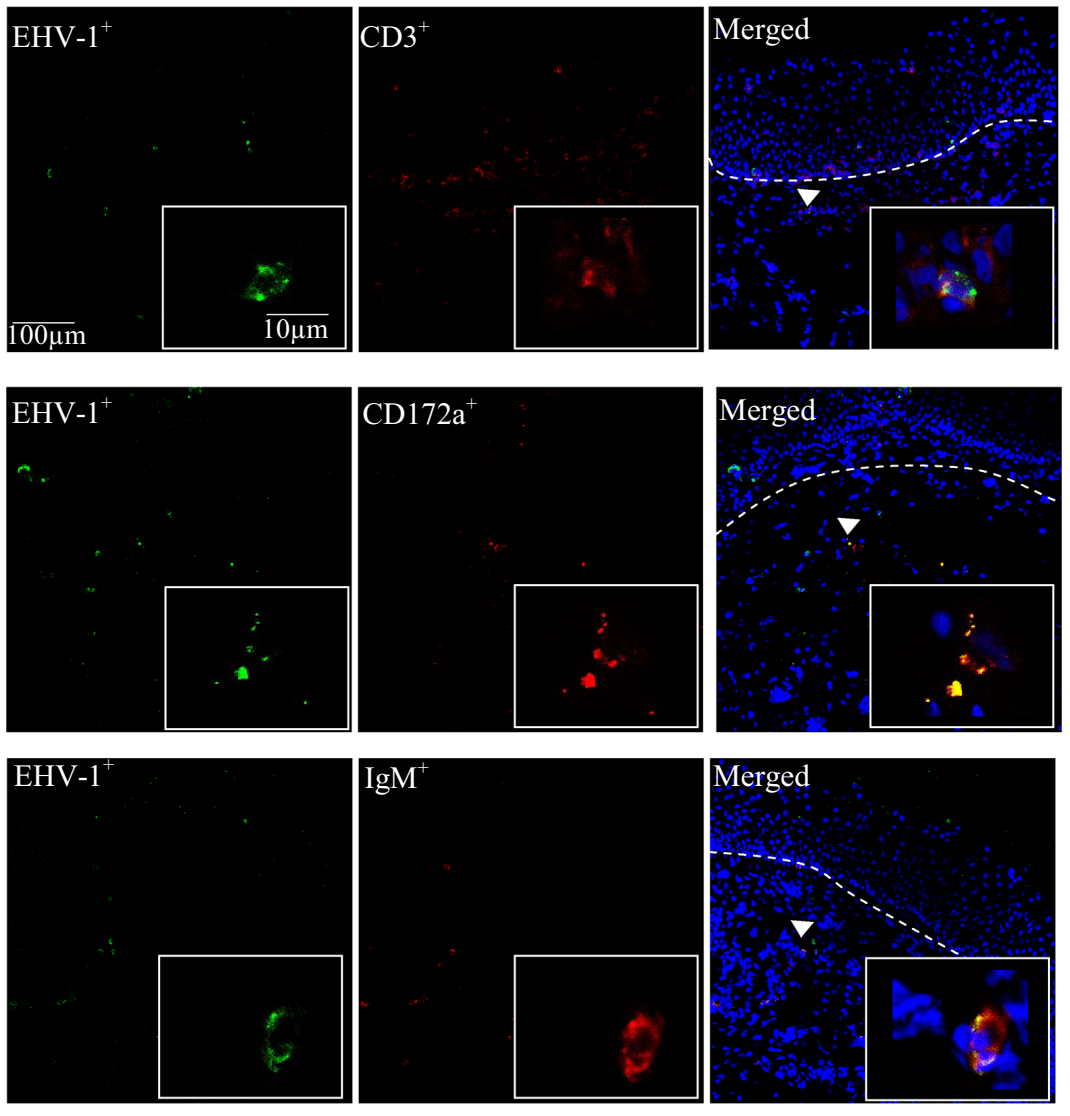
**Representative confocal microscope images of marker positive-EHV-1-infected cells in the vaginal mucosa**. The explants were sectioned (10 µm) and co-immunostained for EHV-1 infected cells (green) and marker positive cells (red). White dotted line indicates the BM. White arrowheads shows double positive cells.

Single EHV-1 and EHV-3-infected cells underneath the BM of the nasal and vaginal mucosa tissues were assessed. In both mucosal tissues, EHV-3-infected cells underneath the BM were totally not detected at all the time points pi. In both mucosae, EHV-1-infected cells was already visible starting from 24 h pi with the neuropathogenic strain and from 48 h with the abortigenic strain (Figure 4).

**Figure 5:**
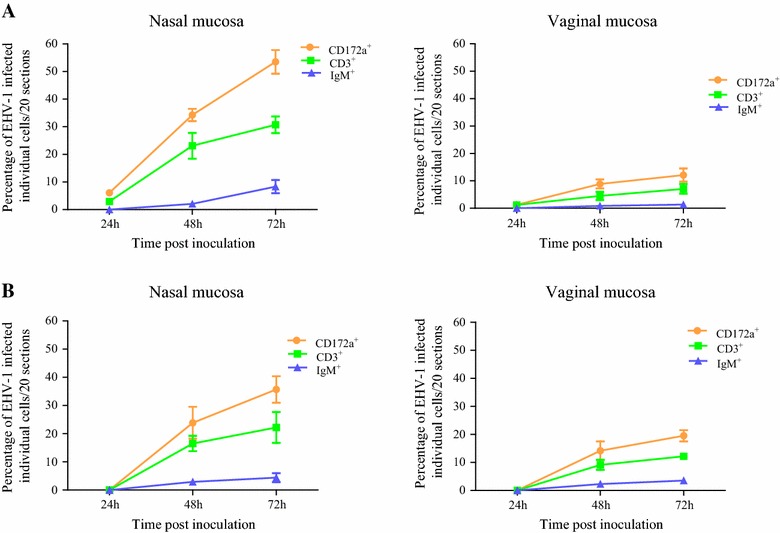
**Percentage of EHV-1-infected individual cells.** The cells were identified as monocytic cells (CD172a^+^), pan T-lymphocytes (CD3^+^) and B-lymphocytes (IgM^+^) per 20 sections of the nasal and vaginal mucosal explant infected with EHV-1 03P37 (**A**) and EHV-1 97P70 (**B**). Lines show the mean ± SD of three independent experiments.

The percentage of abortigenic EHV-1-infected individual monocytic cells was twofold lower in the vaginal mucosa than in the nasal mucosa. Similarly, up to fivefold lower percentage of neuropathogenic EHV-1-infected individual monocytic cells was recorded in the vaginal mucosa than in the nasal mucosa. Regardless of the tissues and strains of EHV-1, CD172a^+^ cells from the monocytic lineage were the predominant cells type infected, followed by CD3^+^ T-lymphocyte. EHV-1 infects IgM^+^ cells (B-lymphocytes) to a much lesser extent (Figure 5).

## Discussion

Neuropathogenic and abortigenic EHV-1 strains, that display different diseases are circulating in the field. The mucosal surface of the upper respiratory tract plays a major role in EHV-1 replication and transmission. Despite several studies conducted on the replication kinetics and characteristics of EHV-1 on the tissues of the upper respiratory tract, the replication efficiency of both pathotypes in the vaginal mucosa was never studied before. EHV-3 has been associated with both genital and respiratory diseases, however the underlying pathogenesis remains poorly understood, due in part to the lack of appropriate models to study virus-host interactions. Therefore, in this study, ex vivo respiratory and vaginal mucosa explant cultures were developed to gain more insight into the replication kinetics and invasion characteristics of the neuropathogenic and abortigenic strains of EHV-1 and to elucidate the pathogenesis of EHV-3 in the nasal and vaginal mucosae, target organs for virus entry and replication. The explants, directly derived from the upper respiratory tract and the vagina of the horses, were maintained in an air–liquid interface for up to 96 h with little change in the viability of the cells as evaluated by ciliary beating and the number of apoptotic cells. The mucosal explant models showed an intact 3D structure and contained all resident mucosal target cells and consequently is ideal to study the virus-host interactions at the site of infection. These explants from slaughterhouse horses replace experimental animals and is, as such, in line with the 3Ds of animal welfare.

In the present study, both types of EHV-l and EHV-3 replicated in a plaquewise manner and spread laterally on the epithelium. The plaques were already present on the epithelium starting from 24 h pi and their sizes significantly increased over time. The plaques did not cross the BM to infect the underlying tissues at all time points pi. However, the invasion via single infected leukocytes in the underlying connective tissue was different. EHV-1 breached the BM and invaded the lamina propria using infected mononuclear immune cells. In contrast, EHV-3 replication was restricted to the epithelial cells, where the virus neither breached the BM in a direct way nor infected individual immune cells to invade the lamina propria. This non-invasive behavior of EHV-3 is comparable with what has been seen with EHV-4 [22], but in contrast with other members of alphaherpesviruses such as pseudorabies virus (PRV) [27], bovine herpesvirus-1 (BoHV-1) [24], herpes simplex virus-1 (HSV-1) [28], canine herpesvirus-1 (CaHV-1) (Yewei Li, unpublished data), feline herpesvirus-1 (FeHV-1) [29] and infectious laryngotracheitis virus (ILTV) [30], which breach the BM and infect the underlying connective tissue. For PRV, it was shown that a cellular serine protease is responsible for this phenomenon [31].

In our study, both strains of EHV-1 types have remarkably higher replication kinetics in the nasal mucosa compared to the vaginal mucosa as evaluated by the number of plaques counted, the size of the plaques, and the amount of infected individual mononuclear immune cells. This suggests that the nasal mucosa is the primary tissue of preference for EHV-1 replication and entry into its host. Both EHV-1 types replicated with comparable kinetics in the nasal mucosa at all time points pi. This result is consistent with the earlier report in the respiratory mucosa explants study [20], where no differences were observed in the replication kinetics between both EHV-1 types. Despite the fact that EHV-1 has a main tropism for the respiratory mucosa, the vaginal mucosa is able to support replication. However, the magnitude of replication in the vaginal mucosa was significantly different between the two EHV-1 strains. The neuropathogenic strain replicated less efficiently than the abortigenic strain and at 72 h pi a lot of non-infected basal cells were observed between the cluster of infected epithelial cells and the BM for the neuropathogenic strain but not for the abortigenic strain. Several reasons may be responsible for this observation. Gryspeerdt et al. [14] reported that after 3 days of infection the production of interferon limits EHV-1 replication in the epithelium of the upper respiratory tract. Maybe the same defense mechanism is activated in the vaginal mucosa. EHV-1 infection induces high antiviral interferon-α levels, which is critical in the host innate immune response [32]. Another possible explanation for the difference in replication efficiency observed between the two EHV-1 strains might be associated with viral genetic factors. Goodman et al. [33] reported that EHV-1 virulence and tissue tropism in the natural host are linked with the function of the DNA polymerase. Indeed, a single point mutation in this enzyme has been claimed to be responsible for the neurotropism. It would now be very interesting to determine if the same point mutation in this enzyme is also determining the level of EHV-1 replication in the vaginal mucosa. It is very well possible that this point mutation is generally affecting the replication of EHV-1 in the different mucosae of the genital tracts (endometrium and vaginal mucosa).

In this study, although we clearly demonstrated that both EHV-1 types are capable of replicating in the mucosa of the vaginal epithelium, the extent of replication is much higher with the abortigenic strain compared with the neuropathogenic strain. Previous reports indicated that EHV-1 has been isolated from male genital organs [34] and is shed with the semen and the sperm cells [35–37]. When we combine the latter reports with our findings, we could propose a possible venereal transmission of EHV-1 via the semen, which was largely ignored before. The venereal transmission have been well documented for other alphaherpesviruses such as EHV-3 [2], BoHV-1 [38, 39], PRV [40] and CaHV-1 [41]. Therefore, our study highlights an important insight that should be further investigated in the field.

In our study, although both the nasal and vaginal mucosae infected by EHV-3, the replication kinetics is significantly different. The sizes of EHV-3-induced plaques are significantly higher in the vaginal mucosa than the nasal mucosa. This replication advantage of EHV-3 in the vaginal mucosa might be associated with the virus tissue tropism, as it has a higher affinity to affect the genital organs under natural conditions. Although, post-coital infection of the EHV-3 is the general route of transmission, non-venereal transmission to the nasal mucosa has been reported via genitonasal contact and contaminated objects [2, 42]. In the current study, EHV-3 replication was restricted to the epithelium mucosae, where the virus neither breached the BM to invade the lamina propria nor infected individual mononuclear immune cells. This localized replication behavior might limit the EHV-3 dissemination via systemic blood circulation. In-vivo EHV-3 infection destroys the epithelium and elicits a vigorous, localized inflammatory response [2] and systemic dissemination of the virus is exceptional [42]. Whether host factors or viral factors inhibit the virus to infect individual immune cells and to invade the underlying tissue is not clear and deserves further investigation.

In the current study, the neuropathogenic strain infects a higher percentage of monocytic cells in the nasal mucosa when compared to the abortigenic strain. This result is in line with the previous report made by Vandekerckhove et al. [20] in the nasal explants study and Gryspeerdt et al. [14] in an in vivo study. However, the neuropathogenic strain infects a lower percentage of cells in the vaginal mucosa than the abortigenic strain of EHV-1. These results suggest that the number of single infected cells may vary with the mucosa type infected with EHV-1. With both EHV-1 types, two-to-five-fold lower percentage of infected monocytic cells were found in the lamina propria of the vaginal mucosa than in that of the nasal mucosa. Infected individual cells underneath the BM were visible already at 24 h pi with the neuropathogenic strain, and at 48 h pi with the abortigenic strain. The monocytic lineage cells, which express surface marker CD172a, were the predominant cell type infected with EHV-1, independent of the strain and tissue. This is in agreement with the previous report in the nasal explants [20], in an in vivo experiment [14], and in peripheral blood mononuclear cells [43, 44]. This cell marker is expressed in equine monocytes, macrophages, dendritic cells and granulocytes [45, 46]. Recently, Baghi et al. [47] reported that isolated equine nasal mucosal CD172a^+^ cells resemble immature dendritic cells. The dendritic cells in the periphery capture and process antigens, express lymphocyte co-stimulatory molecules, migrate to lymphoid organs and secrete cytokines to initiate immune responses [48]. CD3^+^ T- lymphocytes were also an important cell type infected with EHV-1. IgM^+^ cells (B- lymphocytes) were infected to a much lesser extent.

In conclusion, EHV-1 and EHV-3 exhibited different invasion characteristic. Both viruses replicated in a plaquewise manner and spread laterally on the epithelium. EHV-3-induced plaques are restricted to the epithelium of the nasal and vaginal mucosae and the virus neither breaches the BM nor infect individual immune cells at all time points pi. In contrast, EHV-1 invades the underlying connective tissue by infecting mononuclear immune cells. Our ex vivo explant models provided an important new insight that should be further investigated in order to better understand the underlying mechanisms and in vivo relevance.
